# Behavioural Functions and Cerebral Blood Flow in a P301S Tauopathy Mouse Model: A Time-Course Study

**DOI:** 10.3390/ijms22189727

**Published:** 2021-09-08

**Authors:** Faraz Ahmad, Hannah Mein, Yu Jing, Hu Zhang, Ping Liu

**Affiliations:** 1 Department of Anatomy, School of Biomedical Sciences, University of Otago, Dunedin 9016, New Zealand; faraz.ahmad@otago.ac.nz (F.A.); hannah.mein@otago.ac.nz (H.M.); rena.jing@otago.ac.nz (Y.J.); 2 School of Pharmacy, University of Otago, Dunedin 9016, New Zealand; hu.zhang@otago.ac.nz

**Keywords:** tauopathy, PS19 mice, tau, hyperactivity, anxiety, spatial learning and memory, cerebral blood flow, hippocampus

## Abstract

Tauopathies refer to a group of neurodegenerative diseases with intracellular accumulation of hyperphosphorylated and aggregated microtubule-associated protein tau (MAPT) in neurons and glial cells. PS19 mice bearing the MAPT P301S mutation have been used to mimic human frontotemporal lobar degeneration. The present study was designed to systematically investigate how behavioural functions, resting cerebral blood flow (CBF) and tau pathology change in PS19 mice at 2, 4, 6, 8 and 12 months of age in a single study under one experimental condition, allowing for the cumulative assessment of age- and genotype-dependent changes. PS19 mice displayed hyperactivity and reduced anxiety levels with age, early and persistent spatial working memory deficits and reduced resting neocortical CBF. Immunoblotting and immunohistochemistry revealed age-related increases in phosphorylated tau in the brain of PS19 mice. In conclusion, the present study, for the first time, cumulatively demonstrated the time-course of changes in behavioural functions, resting CBF and tau pathology in a P301S tauopathy mouse model through their developmental span. This information provides further evidence for the utility of this model to study neurodegenerative events associated with tauopathy and tau dysfunction.

## 1. Introduction

Tauopathies refer to a group of neurodegenerative diseases with intracellular accumulation of hyperphosphorylated and aggregated microtubule-associated protein tau (MAPT) in neurons and glial cells. Aggregation of hyperphosphorylated tau leads to the formation of paired helical filaments, neurofibrillary tangles (NFTs) and other types of fibrillar deposits, the pathological hallmarks of Alzheimer’s disease (AD), frontotemporal dementia (FTD) and other types of tauopathies [1,2]. Earlier research has shown that multiple pathogenic mutations in MAPT are associated with diverse FTD syndromes, and, contribute to frontotemporal lobar degeneration [3]. Experimentally, both non-transgenic and transgenic animal models of tauopathies are key to understanding the structural, cellular and behavioural effects of abnormal tau. Transgenic mice bearing the MAPT P301 mutation are commonly employed to mimic human frontotemporal lobar degeneration [4,5]. 

PS19 mice are created by overexpressing a human tau form (with four microtubule-binding domains and one N-terminal insert, 4R/1N) with the Pro301Ser mutation [6,7]. These mice show filamentous tau lesions in the brain at 6 months and striking neuronal loss and cortical and hippocampal atrophy at 9–12 months of age [5]. Intriguingly, PS19 mice display synapse loss and synaptic dysfunction in the hippocampus at 3 months, the age point lacking fibrillary tau tangles in the brain [5]. It is therefore likely that the soluble tau species, such as hyperphosphorylated tau oligomers, contribute significantly to the synapse loss and synaptic dysfunction seen in 3 month old PS19 mice [8,9].

PS19 mice bearing the human MAPT P301S mutation display age-related behavioural alterations. A number of studies, for example, have reported impaired motor function, hyperactivity (disinhibited behaviour), reduced anxiety levels, reduced spontaneous alternation and despair behaviour in these tau mice at varying ages of 3–10 months [4,10,11,12,13,14]. PS19 mice also show impaired contextual memory at 6 months [10] and reduced nociceptive thresholds and altered motor coordination at 9 and 10 months of age [4]. Moreover, these tau mice display impaired spatial learning and memory and object recognition memory at 8–9 months [11,15,16], although Sun et al. reported no significant behavioural deficits in the water maze at 9 and 12 months of age [12].

Neurons rely on the brain’s highly dynamic and complex vascular network to assure accurate and adequate distribution of oxygen and glucose. Hence, optimal cerebral blood supply (controlled by cerebral blood flow, CBF) is essential for proper brain function. There is increasing evidence implicating cerebrovascular abnormalities and reduced CBF in the pathogenesis of AD and FTD [17,18,19]. Intriguingly, Park et al. reported a small reduction in resting neocortical and hippocampal CBF in PS19 mice at 2–3 months of age when compared to age-matched WT mice [20]. However, it is unclear whether reduced resting CBF is a transient phenomenon or a long-lasting effect.

The information gathered from various studies shows reduced CBF, synaptic dysfunction and behavioural alterations in PS19 mice with MAPT P301S mutation. The present study was designed to cumulatively determine how behavioural function, resting CBF and tau pathology changed in PS19 mice with age under one experimental condition. Using 2, 4, 6, 8 and 12 months old PS19 mice and their age-matched WT littermates, we systematically assessed the animals’ performance in a battery of behavioural tests including the elevated plus maze, open field, Y-maze and water maze. After completion of the behavioural testing, a real-time microcirculation imager was employed to measure the resting neocortical CBF in PS19 and WT mice at all five age points. Finally, we evaluated age-related changes in tau pathology in PS19 tau mice by focusing on phosphorylated tau species Ser202/Thr205 (AT8), Ser396 (PHF13) and Thr212/Ser214 (AT100) via immunoblotting and immunohistochemistry [21,22].

## 2. Results

### 2.1. Body Weight 

**Figure 1:**
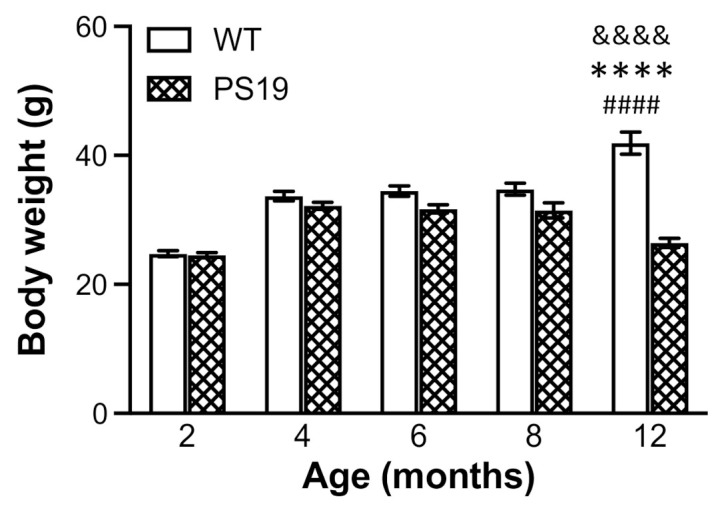
Mean (±SEM) body weights (g) of male wild-type (WT) and PS19 mice at 2, 4, 6, 8 and 12 months of age (*n* = 11–15/genotype/age). ^&&&&^ indicates a significant genotype and age interaction at *p* < 0.0001. **** indicates a significant genotype effect at *p* < 0.0001. ^####^ indicates a significant age effect at *p* < 0.0001.

The animals’ body weights were obtained on the day of sacrifice and tissue collection. Two-way ANOVA revealed significant effects of genotype (F(1,128) = 61, *p* < 0.0001), age (F(4,128) = 33, *p* < 0.0001) and their interaction (F(4,128) = 22, *p* < 0.0001). As illustrated in Figure 1, body weights increased with age, with approximately a 35% increase from 2 to 4 months for both PS19 and WT mice and a 20% increase from 8 to 12 months for WT mice. While both genotype groups had almost identical weights at 2 months of age, PS19 mice displayed body weight reductions at 4 (5%), 6 (10%), 8 (10%) and 12 (37%) months relative to their age-matched WT mice.

### 2.2. Behavioural Data

#### 2.2.1. Elevated plus Maze (EPM) 

**Figure 2:**
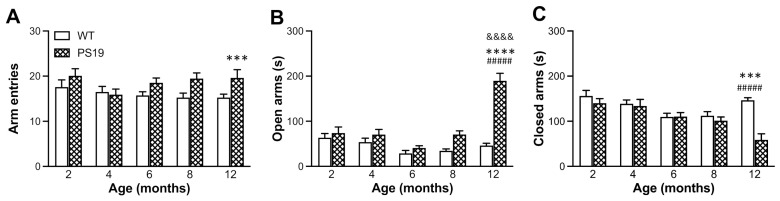
Mean (±SEM) total number of arm entries (**A**) and time (s) spent in open arms (**B**) and closed arms (**C**) of male wild-type (WT) and PS19 mice at 2, 4, 6, 8 and 12 months of age in the elevated plus maze test (*n* = 11–15/genotype/age). ^&&&&^ indicates a significant genotype and age interaction at *p* < 0.0001. * indicates a significant genotype effect at *** *p* < 0.001 or **** *p* < 0.0001. ^#####^ indicates a significant age effect at *p* < 0.0001.

All animals were tested in the EPM for a duration of 5 min. When the total number of arm entries was analysed, there was a significant effect of genotype (F(1,123) = 11.33, *p* = 0.001) but not age or interaction (both F ≤ 1), with more entries in 6 (18%), 8 (28%) and 12 (28%) months old PS19 mice relative to their age-matched WT controls (Figure 2A). Regarding the time spent in open arms (Figure 2B), ANOVA revealed significant effects of genotype (F(1,123) = 54.38, *p* < 0.0001), age (F(4,123) = 24.79, *p* < 0.0001) and their interaction (F(4,123) = 19.66, *p* < 0.0001), with 106% and 310% more time in PS19 mice at 8 and 12 months, respectively, when compared to their age-matched WT mice. When the time spent in closed arms was analysed, we observed significant effects of genotype (F(1,123) = 14.31, *p* = 0.0002), age (F(4,123) = 7.87, *p* < 0.0001) and their interaction (F(4,123) = 7.11, *p* < 0.0001), with gradually reduced time from 2 to 8 months in both groups and a 60% reduction in PS19 mice relative to WT controls at 12 months of age (Figure 2C). Collectively, these data demonstrate the hyperactivity and reduced anxiety levels in PS19 mice in an age-dependent manner.

#### 2.2.2. Open Field 

**Figure 3:**
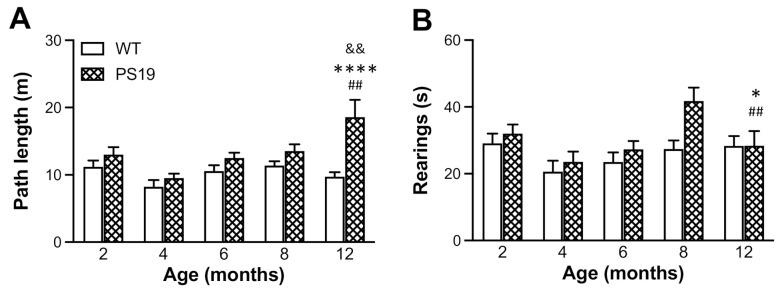
Mean (±SEM) path length (m, **A**) and duration of rearings (**B**) of male wild-type (WT) and PS19 mice at 2, 4, 6, 8 and 12 months of age in the open field test (*n* = 11–15/genotype/age). ^&&^ indicates a significant genotype and age interaction at *p* < 0.01. * indicates a significant genotype effect at * *p* < 0.05 or **** *p* < 0.0001. ^##^ indicates a significant age effect at *p* < 0.01.

All animals were tested in the open field for a period of 5 min following the EPM test. When the total path length travelled in the apparatus was analysed (Figure 3A), ANOVA revealed significant effects of genotype (F(1,128) = 17.76, *p* < 0.0001), age (F(4,128) = 4.69, *p* = 0.0014) and their interaction (F(4,128) = 3.68, *p* = 0.007). PS19 mice generated 15–20% and 90% longer path lengths at 2–8 and 12 months, respectively, when compared to their age-matched WT controls. Regarding the duration of rearings (Figure 3B), there were significant effects of genotype (F(1,128) = 5.54, *p* = 0.02) and age (F(4,128) = 4.23, *p* = 0.003) but not their interaction (F(4,128) = 1.58, *p* = 0.18), with 50% more rearings in 8 months old PS19 mice relative to age-matched WT mice. These data show the hyperactivity and increased level of exploration in PS19 mice in an age-specific manner.

#### 2.2.3. Y-Maze 

**Figure 4:**
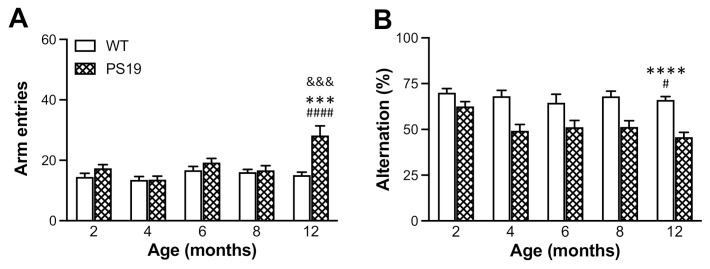
Mean (±SEM) total number of arm entries (**A**) and percentage of spontaneous alternation (**B**) of male wild-type (WT) and PS19 mice at 2, 4, 6, 8 and 12 months of age in the Y-maze test (*n* = 11–15/genotype/age). ^&&&^ indicates a significant genotype and age interaction at *p* < 0.001. * indicates a significant genotype effect at *** *p* < 0.001 or **** *p* < 0.0001. ^#^ indicates a significant age effect at ^#^ *p* < 0.05 or ^####^ *p* < 0.0001.

The animals’ spontaneous alternation behaviour was assessed in the Y-maze apparatus. Regarding the total number of arm entries (Figure 4A), ANOVA revealed significant effects of genotype (F(1,126) = 14.22, *p* = 0.0002), age (F(4,126) = 6.92, *p* < 0.0001) and their interaction (F(4,126) = 5.84, *p* = 0.0002), with 86% more entries in 12 month old PS19 mice relative to age-matched WT mice. When the percentage of alternations was analysed (Figure 4B), there were significant effects of genotype (F(1,126) = 58.26, *p* < 0.0001) and age (F(4,126) = 2.97, *p* = 0.02) but not their interaction (F(4,126) = 1.26, *p* = 0.29), with PS19 mice displaying 10% and 20–30% reductions at 2 and 4–12 months, respectively, when compared to their age-matched WT controls. These results further support age-related hyperactivity and demonstrate early and long-lasting reduction in spontaneous alternations (hence, working memory deficits) in PS19 mice.

#### 2.2.4. Water Maze 

**Figure 5:**
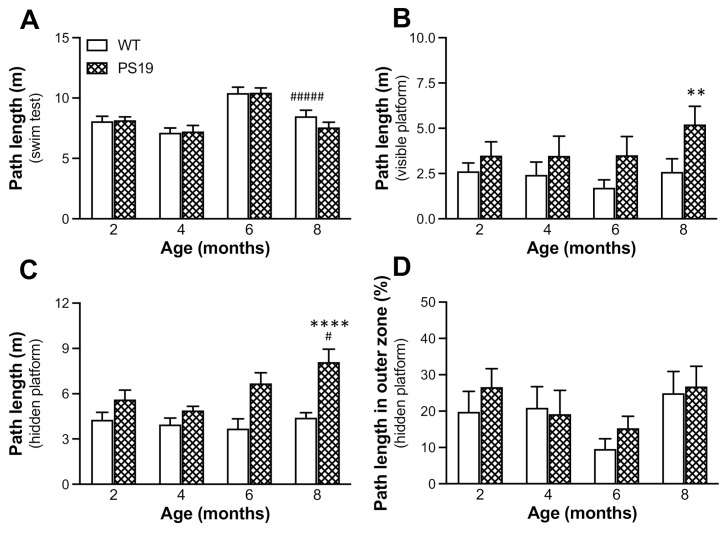
Mean (±SEM) path length (m, **A**–**C**) and percentage of path length in the outer zone (**B**) of male wild-type (WT) and PS19 mice at 2, 4, 6, 8 and 12 months of age in the swim test (**A**); cued navigation trial (**B**; visible platform) and place navigation trials (**C**,**D**; hidden platform) of the water maze test (*n* = 11–15/genotype/age). * indicates a significant genotype effect at ** *p* < 0.01 or **** *p* < 0.0001. ^#^ indicates a significant age effect at ^#^ *p* < 0.05 or ^#####^ *p* < 0.0001.

Since PS19 mice display motor deficits with age [4], we first evaluated any compromises in swimming in animals used in the present study. During a single 60 s swim test without platform, PS19 mice at 12 months of age showed obvious motor compromises and were therefore excluded from the water maze tests. For PS19 and WT mice at 2, 4, 6 and 8 months age points, we found a significant effect of age (F(3,98) = 21.54, *p* < 0.0001) but not genotype or interaction (both F < 1), with a greater path length at 6 months relative to other age points for both PS19 and WT mice (Figure 5A).

All animals at four age points were then tested in a working memory version of the water maze task for two consecutive days, with the first day treated as habituation. When the data obtained from the second day of testing were analysed, there was a significant effect of genotype (F(1,98) = 7.31, *p* = 0.008) but not age or interaction (both F ≤ 1) in the path length measurement during the cued navigation trial (trial 1) in the presence of a visible platform (Figure 5B). PS19 mice generated 33% (2 months), 43% (4 months) and 100% (6 and 8 months) longer path lengths for finding the platform relative to their age-matched WT controls. All animals were then trained to find a hidden platform presented in the same place during trials 2–6. When the mean path length of the five trials was analysed (Figure 5C), ANOVA revealed significant effects of genotype (F(1,98) = 27, *p* < 0.0001) and age (F(3,98) = 3.29, *p* = 0.02) but not their interaction (F(3,98) = 2.31, *p* = 0.08), with age-related increases primarily in PS19 mice. Moreover, PS19 mice generated 20–30% (2 and 4 months) and 80% (6 and 8 months) longer path lengths to reach the hidden platform relative to their age-matched WT controls. We further analysed thigmotaxic swimming during trials 2–6 (Figure 5D) and found no significant effects of genotype (F < 1), age (F(3,98) = 2.54, *p* = 0.061) and their interaction (F < 1). Taken together, these results show impaired performance in PS19 mice in searching for both visible and hidden platforms in an age-related manner.

### 2.3. Resting Neocortical CBF

**Figure 6:**
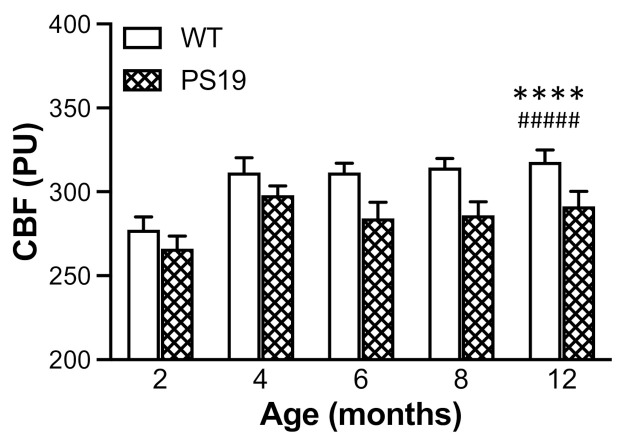
Mean (±SEM) resting cerebral blood flow (CBF) in the neocortex (presented as perfusion unit, PU) of male wild-type (WT) and PS19 mice at 2, 4, 6, 8 and 12 months of age (*n* = 11–15/genotype/age). **** indicates a significant genotype effect at *p* < 0.0001. ^#####^ indicates a significant age effect at *p* < 0.0001.

Using a real-time microcirculation imager, we measured the resting neocortical CBF in PS19 and WT mice at all five age points. ANOVA revealed significant effects of genotype (F(1,128) = 19.64, *p* < 0.0001) and age (F(4,128) = 6.63, *p* = 0.0001) but not their interaction (F < 1), with a 5–10% reduction in PS19 mice relative to WT mice across all five age points (Figure 6).

### 2.4. Tau Pathology

We assessed tau pathology, such as phosphorylated tau species Ser202/Thr205 (AT8), Ser396 (PHF13) and Thr212/Ser214 (AT100), in a subset of PS19 mice at five age points and their age-matched littermate WT controls using immunoblotting (i.e., AT8, PHF13 and AT100) and immunohistochemistry (AT8 only). 

**Figure 7:**
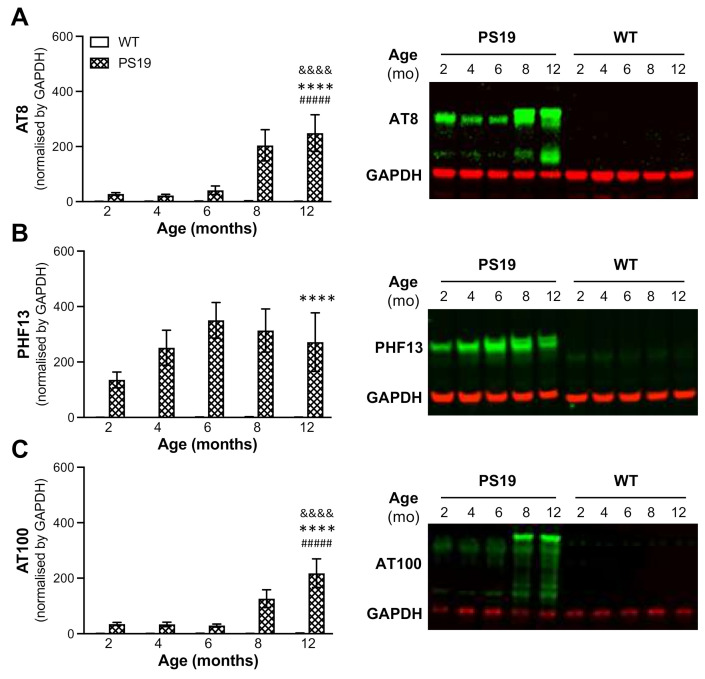
Mean (±SEM) protein levels of phosphorylated tau species Ser202/Thr205 (AT8; **A**), Ser396 (PHF13; **B**) and Thr212/Ser214 (AT100; **C**) in the hippocampus of male wild-type (WT) and PS19 mice at 2, 4, 6, 8 and 12 months (mo) of age (*n* = 11–15/genotype/age), with example immunoblots of AT8, PHF13, AT100 and GAPDH (data were normalised by GAPDH). ^&&&&^ indicates a significant genotype and age interaction at *p* < 0.0001. **** indicates a significant genotype effect at *p* < 0.0001. ^##^^###^ indicates a significant age effect at *p* < 0.0001.

The levels of AT8, PHF13 and AT100 data in the hippocampal lysates were determined using immunoblotting, and the data were normalised by housing keeping protein, glyceraldehyde 3-phosphate dehydrogenase (GAPDH). Regarding AT8 (Figure 7A), ANOVA revealed significant effects of genotype (F(1,60) = 36.36, *p* < 0.0001), age (F(4,60) = 7.51, *p* < 0.0001) and their interaction (F(4,60) = 7.41, *p* < 0.0001). While AT8 expression was almost negligible in WT mice at all age points, PS19 mice displayed relatively lower levels at 2, 4 and 6 months; however, there were marked increases (7–9-fold) at 8 and 12 months of age. Regarding PHF13 (Figure 7B), there was a significant effect of genotype (F(1,60) = 66.31, *p* < 0.0001) but not age or interaction (both F = 1). Again, PHF13 expression was almost negligible in WT mice at all ages. PS19 mice, however, displayed a relatively high level of PHF13 at 2 months, with further increases, reaching saturation at 6 months of age. For AT100 (Figure 7C), ANOVA revealed significant effects of genotype (F(1,60) = 50, *p* < 0.0001), age (F(4,60) = 9.2, *p* < 0.0001) and their interaction (F(4,60) = 9.0, *p* < 0.0001). Similar to AT8, AT100 expression was almost negligible in WT mice at all five age points. PS19 mice displayed relatively lower levels at 2, 4 and 6 months; however, there were marked increases (4–7-fold) at 8 and 12 months of age. These immunoblotting results confirmed age-related increases in the levels of phosphorylated tau species Ser202/Thr205 (AT8), Ser396 (PHF13) and Thr212/Ser214 (AT100) in the hippocampus of PS19 mice.

**Figure 8:**
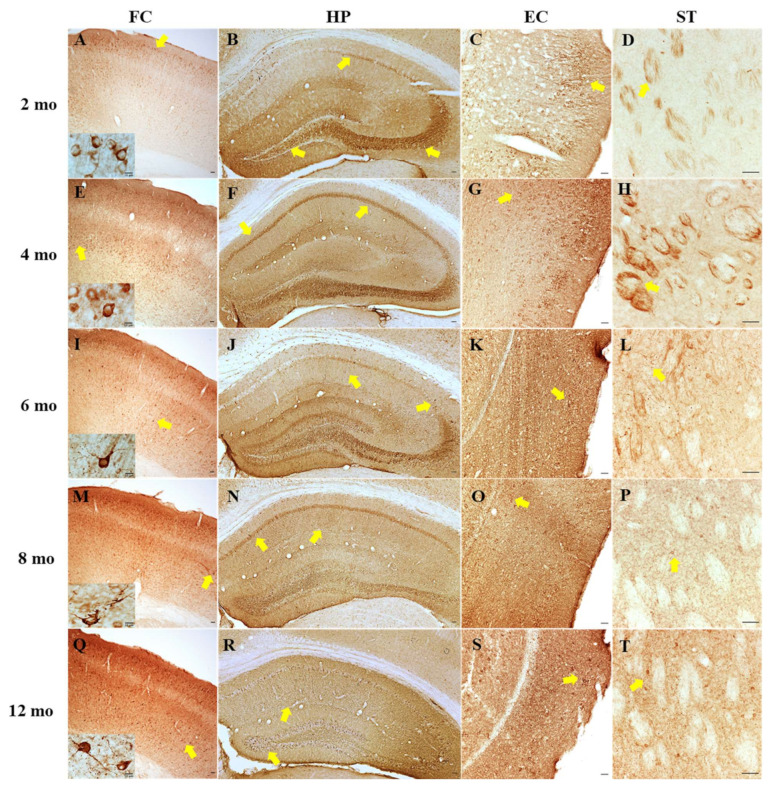
Immunohistochemistry with the AT8 antibody showing phosphorylated tau (Ser202/Thr205) in the frontal cortex (FC; **A**,**E**,**I**,**M**,**Q**), hippocampus (HP; **B**,**F**,**J**,**N**,**R**), entorhinal cortex (EC; **C**,**G**,**K**,**O**,**S**) and striatum (ST; **D**,**H**,**L**,**P**,**T**) of PS19 mice at 2 (**A**–**D**), 4 (**E**–**H**), 6 (**I**–**L**), 8 (**M**–**P**) and 12 (**Q**–**T**) months of age. Scale bars: 50 µm for each panel.

We then assessed the AT8 immunoreactive profile in the frontal cortex, hippocampus, entorhinal cortex and striatum. Immunohistochemistry revealed no positive AT8 staining in the brain of WT mice or with the omission of the primary antibody (data not shown). However, there were clear age-related increases in AT8 immunoreactivity in PS19 mice in all brain regions examined. At 2 months of age, AT8 immunoreactivity was most predominant across the frontal cortex, particularly layers II/III (Figure 8A). Perikaryal and axonal staining was also strong in the pyramidal layer of hippocampal CA3 sub-region (Figure 8B) and layers II/III of the entorhinal cortex (Figure 8C), with less in the CA1/CA2 pyramidal layers and both the polymorph and granule layers of the dentate gyrus. In the striatum, AT8 immunoreactivity was primarily evident in the fibres (Figure 8D). At 4 months of age, AT8 immunoreactivity was markedly increased, particularly in layer V of the frontal cortex (Figure 8E) and the CA1/2 pyramidal layers (Figure 8F), along with the entorhinal cortex (Figure 8G) and striatum (Figure 8H). At 6 months of age, dendritic staining was more prominent throughout all of the investigated brain regions, and tau neuronal lesions were present sparsely in the frontal cortex (Figure 8I), hippocampus (Figure 8J) and entorhinal cortex (Figure 8K). At 8 months of age, further increased staining of the neuropil and neuronal lesions was evident in the frontal cortex (Figure 8M), hippocampus (Figure 8N) and entorhinal cortex (Figure 8O). Whereas, in the striatum, perikaryal immunoreactivity was more abundant (Figure 8P). At 12 months of age, atrophy was apparent in the hippocampus (Figure 8R) and entorhinal cortex (Figure 8S). Perikaryal staining was sparse with intense staining of the neuropil and numerous neuronal inclusions in the frontal cortex (Figure 8Q), hippocampus (Figure 8R) and entorhinal cortex (Figure 8S). In comparison, however, the striatum had increased immunoreactive soma with the fibres less intensely stained (Figure 8T).

## 3. Discussion

PS19 mice harbouring the human MAPT P301S mutation elicit age-related accumulation of phosphorylated tau and NFT formation. These animals show hippocampal synapse loss and synaptic dysfunction at 3 months of age, the appearance of NFTs from 6 months and neuronal loss and hippocampal atrophy at 9–12 months [23]. Intriguingly, PS19 mice also display a small reduction in resting neocortical and hippocampal CBF at 2–3 months of age [20]. Moreover, it has been shown in different studies that these mice display age-related behavioural alterations at various ages from 3 to 12 months [4,10,11,12,13,14,15,16]. For the first time, we systematically and cumulatively assessed behavioural function, resting neocortical CBF and tau pathology in PS19 mice at the age points of 2, 4, 6, 8 and 12 months in a single study under one experimental condition.

The animals’ general health conditions were monitored during the entire course of the study. While WT mice continued to gain weight with age until 12 months, body weight in PS19 mice appeared to reach the maximum at 4 months, become flat at 4–8 months and then decline at 12 months by approximately 17%. At 2 months of age, the body weight in both genotype groups was almost identical. However, PS19 mice displayed weight reductions at 4 (5%), 6 (10%), 8 (10%) and 12 (37%) months. López-González et al. observed lower body weight in PS19 mice from 3 months of age relative to WT mice, which was more evident from the age of 7 months [4]. Recently, Sun et al. reported a significant difference in body weight between PS19 and WT mice at nine age points ranging from 3.5 to 12 months [12]. Taken together, PS19 mice show age-related reduction in body weight.

EPM is one of the most commonly used tests to evaluate anxiety-like behaviour in rodents based on their natural aversion for open and elevated areas [24,25]. On the other hand, open field is a general behavioural test to assess animals’ locomotion and exploratory activity based on their natural conflict between exploration of and aversion against bright open areas in a novel environment [26]. Earlier research has shown reduced anxiety levels and hyperactivity (disinhibited behaviour) as one of the most striking features of PS19 mice [4,10,11]. Consistent with these earlier reports, our PS19 mice (particularly for those at older ages) made more arm entries and spent more time in the open arms in the EPM and generated greater path lengths in the open field apparatus. Exploratory behaviour (such as rearings) has often been used to assess animals’ ability to integrate spatial features into a representation of a novel environment [27], and it is important for both allothetic and idiothetic navigation [28]. In the present study, the duration of rearings significantly increased in PS19 mice at 8 months of age, which might be attributed to the hyperactivity to a certain extent. The relatively lesser time spent in wall-supported rearings in 12 months old PS19 mice may be due to their motor deficits and, hence, an aversion for the challenging exploratory posture. 

Spontaneous alternation behaviour in the Y-maze is a measure of spatial working memory in rodents based on their innate curiosity to explore previously unvisited arms [29,30]. PS19 mice at 3–4 months of age displayed a significantly reduced percentage of spontaneous alternation (approximately 12%) but an increased number of arm entries in the Y-maze, indicating impaired working memory and hyperactivity in mice with MAPT P301S mutation [10]. In the present study, we observed a very strong genotype effect in the percentage of spontaneous alternation measurement. Relative to age-matched WT controls, PS19 mice displayed 10% and 20–30% reductions in spontaneous alternation at 2 and 4–12 months, respectively. While PS19 mice at 12 months of age made 86% more arm entries relative to WT mice, hyperactivity was not apparent in these tau mice at younger ages in the Y-maze test. Hence, while the data demonstrate an early and long-lasting change in spontaneous alternation behaviour in PS19 mice, our results also suggest that this working memory deficit is independent of hyperactivity.

The water maze is a commonly used test to assess spatial learning and memory in rodents [31]. Normally, animals are trained to find a hidden platform in a fixed position over a number of days, and their memory of the platform location is evaluated via a probe test in the absence of the platform. Earlier research has assessed how PS19 mice perform in this type of reference memory version of the water maze task, however, with mixed results. For example, some studies reported spatial learning and memory deficits in the water maze task in PS19 mice at 6 [10], 8–9 [11] and 10 [32] months of age. However, Sun et al. found no significant behavioural deficits in the water maze test in PS19 mice at 9 and 12 months of age [12], although age-related increases in exploratory activity were evident. In the present study, PS19 and WT mice at 2–8 months of age were tested in a working memory version of the water maze task [33]. Animals at four age points were trained to find a visible (trial 1) and hidden (trials 2–6) platform with the location kept the same for all trials on each day but varied between two consecutive days of testing, and the first day was treated as habituation. PS19 mice at 12 months of age were excluded from the test because of their motor compromises. We found a significant genotype effect in terms of the path length to reach the visible platform, and PS19 mice were markedly impaired in searching for the hidden platform (although in the same location) in an age-related manner. Because there were no significant differences in path length during the swimming test and thigmotaxic swimming in five place navigation trials between the two genotype groups at all four age points, our results demonstrate clear age-associated performance impairments in this working memory version of the water maze task in PS19 mice.

Using a real-time microcirculation imager, for the first time, the present study measured the resting neocortical CBF in both PS19 and WT mice at 2, 4, 6, 8 and 12 months of age. Intriguingly, there was a 5–10% reduction in the resting CBF in PS19 mice at all five age points relative to their age-matched WT mice, although the effects were more pronounced at later age points. Of note, Park et al. observed a similar level of reduced resting cortical CBF as well as impaired neurovascular coupling response in the barrel cortex following facial whisker stimulation in PS19 mice at 2–3 months of age [20]. Taken together, these two studies demonstrate early and long-lasting cerebrovascular dysfunction in PS19 mice with the human MAPT P301S mutation. There is evidence suggesting that neurofibrillary lesions can impact on brain endothelial cell biology, altering the integrity of the brain’s microvasculature [34]. Given the fact of altered CBF and neurovascular coupling in PS19 mice prior to the presence of neurofibrillary lesions ([20], present study), soluble tau oligomers may have a significant impact on the structure and/or function of cerebral vasculature, which remains to be determined in future research.

It has been shown that human tau in PS19 mice begins to accumulate at 3 months of age and continues increasing until 12 months [12]. The present study further determined tau hyperphosphorylation in the hippocampus of PS19 mice at all five age points using AT8, PHF13 and AT100 antibodies via the immunoblotting approach. Consistent with early research, we found dramatic age-related increases in the level of phosphorylated tau at Ser202/Thr205 (AT8) and Thr212/Ser214 (AT100) at 8 and 12 months age points and an overall high level of phosphorylated tau at Ser396 (PHF13) at all five age points with a peak at 6 months. Moreover, immunohistochemistry revealed clear age-related increases in AT8 immunoreactivity in the frontal cortex, hippocampus, entorhinal cortex and striatum of PS19 mice at five age points in a region-specific manner. At 12 months of age, atrophy was apparent in the hippocampus and entorhinal cortex of PS19 mice, with intense perikaryal AT8 staining and numerous neuronal tau inclusions in these regions and the frontal cortex. Taken together, our results confirmed age-related increases in phosphorylated tau in the brain were evident in the PS19 mice in the present study, which displayed CBF reduction and behavioural deficits.

In summary, the present study determined behavioural functions, resting neocortical CBF and tau pathology in male PS19 mice at 2–12 months of age under one experimental condition. Specifically, we found hyperactivity/disinhibition, reduced anxiety levels, spatial working memory deficits and reduced resting cortical CBF in PS19 mice. While some of these changes were age-dependent, spatial working memory deficits in the Y-maze and water maze and resting CBF reduction appeared to be early and long-lasting. Moreover, immunoblotting and immunohistochemistry revealed age-related increases in phosphorylated tau in the brain of PS19 mice, which likely underlie the observed behavioural and CBF changes. It has been well documented that synapse function is key to memory and cognition [35,36,37]. Interestingly, we have demonstrated the deficits in chemically induced long-term potentiation in cortical synaptosomes of PS19 mice at 8 months of age [38]. The time-course of changes in synaptic function remains to be determined in future research. Park et al. suggest PSD-95-neuronal nitric oxide synthase uncoupling as a primary contributor of alterations in cerebrovascular homeostasis seen in 2–3 month old PS19 mice [12]. L-arginine, a substrate of neuronal nitric oxide synthase, is a semi-essential amino acid with a number of bioactive molecules [39], and polyamines (its downstream metabolites) are critically involved in microtubule assembly and stabilisation [40,41,42,43]. Intriguingly, PS19 mice displayed early and/or prolonged alterations in polyamines and their precursors with age in the frontal cortex and hippocampus [44]. The functional significance of these changes and other neurochemical and/or molecular mechanisms underlying cognitive decline and microvascular abnormalities in PS19 mice with tau mutation remains to be explored in the future.

## 4. Materials and Methods

### 4.1. Animals

Male P301S tau transgenic (PS19) mice (B6; C3-Tg(Prnp-MAPT*P301S)PS19Vle/J; stock number: 008169; Jackson Laboratory) and C57BL/6J female mice were crossed to produce the offspring of PS19 mice and wild-type (WT) littermates (confirmed by tail tip genotyping). It has been shown that male PS19 mice often display more consistent phenotype relative to females (Jackson Laboratory; Sun et al., 2021). Therefore, male PS19 mice and their WT littermates at 2, 4, 6, 8 and 12 months of age (*n* = 11–15/genotype/age) were used in the present study. Animals were group housed in 15 × 20 × 38 cm^3^ polypropylene individually ventilated cages, maintained on a 12 h light/dark cycle regime (light on at 07:00), and provided ad libitum access to food and water. All mice were individually identified by ear perforation nomenclature, and their body weights and general health conditions were closely monitored. Animals were acclimatised with the facility and experimenters for at least 5 days. All experimental procedures were carried out in accordance with the regulations of the University of Otago Committee on Ethics in the Care and Use of Laboratory Animals and New Zealand legislation (Protocol code AUP-18-95; approved on 14 September 2018). Every attempt was made to reduce the number of animals used and to minimise their suffering.

### 4.2. Chemicals and Antibodies 

Phosphatase inhibitor cocktail IV (ab201115) and antibody against GAPDH (ab181602) were purchased from Abcam. Primary antibodies against phosphorylated tau at Ser396 (PHF13; sc-32275), Ser202/Thr205 (AT8; MN1020) and Thr212/Ser214 (AT100; MN1060) were procured from Santa Cruz Biotechnology and Thermo Scientific, respectively. Secondary antibodies IRDye^®^ 800 CW goat anti-mouse (926-32210) and IRDye^®^ 680 RD goat anti-rabbit (926-68071) were sourced from LI-COR Biosciences. Immunohistochemical biotinylated link and streptavidin/HRP complex solutions (LSAB2 System-HRP Kit, K0675) were from Dako. Protease inhibitors pepstatin A (P5318), leupeptin hydrochloride (L9783) and phenylmethanesulfonylfluoride (PMSF; P7626), 3,3’-diaminobenzidine and H_2_O_2_/urea tablets (D4293-50SET) and DPX mounting media (44581) were from Sigma–Aldrich. Other reagents used in this study were of analytical grade and procured from either Thermo Fisher Scientific or Sigma–Aldrich.

### 4.3. Behavioural Procedures 

Animals at all five age points received a battery of behavioural tests in the EPM, open field, Y-maze and water maze for 4 consecutive days. The behavioural testing was conducted in a windowless room with four 60 W light bulbs placed in the corners. During the tests, the animals’ behaviour was recorded via a video camera mounted in the centre of the room at ceiling height, and the footage was then analysed by TopScan software (Clever Sys Inc., Reston, VA, USA). Extramaze cues consisted of laboratory furniture, lights, prominent visual cues on the walls and the location of the experimenter, and they were held constant for all animals throughout the entire study. For each apparatus, the order of animals tested was counterbalanced between PS19 and WT mice at each age point, and the experimenter was blind for the grouping information.

#### 4.3.1. Elevated Plus Maze (Day 1) 

The EPM apparatus was white in colour with four arms (29 × 6 cm). There were two open arms (surrounded by 1 cm clear Plexiglas) and two closed arms (surrounded by 15 cm high clear Plexiglas) connected to a small central platform (6 × 6 cm). The EPM was elevated 60 cm above the floor, and the positions of the open and closed arms were kept constant for all animals. PS19 and WT mice at each age point were individually placed in the central platform of the maze heading towards a closed arm and allowed to explore the apparatus freely for 5 min. The duration of time spent in and the number of entries to the open and closed arms were analysed. An arm entry was scored when all four paws were in the arm.

#### 4.3.2. Open Field (Day 1) 

The open field apparatus was a 40 × 40 cm white Plexiglas chamber with identical 20 cm high walls and an open top elevated 60 cm above the floor. After the completion of the EPM (120–180 min), animals were individually placed into the chamber at the same position and orientation and allowed to explore the apparatus freely for 5 min. The total path length travelled during the entire testing period and the duration of rearings were analysed using the TopScan tracking system.

#### 4.3.3. Y-Maze (Day 2)

The Y-maze was shaped like a Y and made of white Plexiglas with a 120° angle between each of the three arms (40 × 6 × 13 cm^3^). The apparatus was elevated 60 cm above the floor, and the positions of the arms were kept constant for all animals during the test. Each arm was assigned either A, B or C. PS19 and WT mice at each age point were individually placed at the centre of the maze facing arm “A” and allowed to freely explore the apparatus for a period of 5 min. The number and the sequence of arm entries were recorded. Again, an arm entry was scored when all four paws were in the arm. Alternation behaviour was defined as consecutive entries into all three arms (i.e., ABC, CAB or BCA but not ABA) [29,45]. The percentage of spontaneous alternations was calculated as the ratio of the actual number of alternations to the possible number (defined as the total number of arm entries minus two) multiplied by 100, i.e., % alternation = ((number of alternations)/(total number of arm entries – 2)) × 100. The percentage of spontaneous alternation was measured as an index of working memory, whereas the total number of arm entries reflected the level of locomotor activity.

#### 4.3.4. Water Maze (Days 2–4)

The water maze pool was a white plastic circular tank measuring 100 cm in diameter and 35 cm in height. It was filled daily with water to a level 15 cm below the top, and the temperature was maintained at 21 ± 1 °C. Four points on the edges of the tank were designated as north (N), south (S), east (E) and west (W), allowing the pool to be divided into four quadrants (i.e., NE, SW, NW and SE). It has been shown that PS19 mice display age-related motor deficits [4,5,46]. After completion of the Y-maze test on day 2, an animal’s swimming ability was therefore tested by placing the mouse into the pool facing the wall from the starting point N and allowing it to swim freely for a duration of 60 s. PS19 mice at 12 months of age displayed difficulties in swimming and were therefore excluded from the water maze test.

On days 3 and 4, PS19 and WT mice at each age point were then tested in a working memory version of the water maze task [33]. On each day, there were 6 trials for each mouse with a 60 s interval between trials. For the first trial, animals were trained to find a visible platform (6 cm in diameter) 1 cm above the water with the edge attached to a black upright plastic tag (5 × 3 cm^2^). For the subsequent 5 trials (trials 2–6), animals were trained to find the same platform placed in the same position 2 cm below the water. For each trial, the mouse was gently placed into the pool facing the wall and allowed to swim freely in search of the platform for a maximum of 60 s and to stay on the platform for 10 s before being removed, dried and placed into a holding box. If the mouse did not find the platform within 60 s of being placed into the pool, it was immediately placed on or, if near, guided to the platform for 10 s before being returned to the holding box. The platform location and starting points (i.e., N, S, W, E) were pseudo-randomly selected and changed on each day but were kept the same for all of the animals.

Following completion of the water maze test, several performance variables were analysed offline using the TopScan tracking system. The distance (i.e., path length) the mouse swam during the 60 s period of the swim test or from the starting point to reach the visible/hidden platform was measured. Moreover, the degree of thigmotaxic swimming (i.e., swimming close to the wall) was quantified by dividing the maze into two circles and measuring the time spent in the outer 10% of the pool.

### 4.4. Measurement of Resting Cerebral Blood Flow

After a 2 day washout period following the behavioural testing, CBF was measured in all mice using a real-time microcirculation imager (PeriCam PSI HRsystem, Perimed, Sweden), which operates using laser speckle contrast analysis technology and measures blood perfusion in arbitrary PUs [47]. Mice were anesthetised with isoflurane (2.5% for initiation and 1% for maintenance) and positioned on a stereotaxic apparatus. The animals’ body temperatures were maintained at 37 °C using a heating pad. The skull was then exposed by a mid-line incision, and the imager was positioned approximately 10 cm above the skull. Blood perfusion was recorded for a period of 10 min, and the last 5 min of recording (more stable) was taken for the CBF analysis using the Pimsoft software (Perimed, Sweden). Again, the order of testing was counterbalanced between the two genotype groups at each age point, and the experimenter was blind to the grouping information.

### 4.5. Hippocampal Tissue Collection 

Immediately after completion of the CBF measurement, animals were transcardially perfused with ice-cold saline, and the brains were rapidly removed and left in cold saline (4 °C). The whole hippocampus from each hemisphere was freshly dissected on ice, snap-frozen on dry ice and then stored at −80 °C for immunoblotting [33,38,47].

One pair of PS19 and WT mice at each age point were transcardially perfused with ice-cold saline followed by 4% phosphate-buffered paraformaldehyde. Whole brains were removed, fixed in 4% paraformaldehyde for a further 24 h, and then left in 30% sucrose at 4 °C until they sunk to the bottom of the container. The brains were embedded in an optimal cutting temperature compound, rapidly frozen in isopentane cooled in liquid nitrogen and then stored at −80 °C until sectioning for immunohistochemistry.

### 4.6. Immunoblotting 

Immunoblotting was performed to evaluate age-related changes of phosphorylated tau species at Ser202/Thr205, Ser396 and Thr212/Ser214 in the hippocampus using a subset of PS19 mice and their age-matched WT mice. The whole hippocampal tissues were lysed in 50 mM Tris-HCl (pH 7.4) supplemented with a cocktail of protease (15 µm pepstatin A, 2 µm leupeptin and 10 µm PMSF) and phosphatase inhibitors by sonication on ice, followed by centrifugation at 12,000× *g* for 10 min at 4 °C. The supernatants were obtained for immunoblotting to measure the levels of phosphorylated tau at Ser202/Thr205, Ser396 and Thr212/Ser214 using the AT8, PHF13 and AT100 antibodies, respectively.

The protein concentration in each supernatant was determined by the Bradford assay and then equalised to 2 mg/mL. Brain tissue homogenates were mixed with gel loading buffer (containing 50 mM Tris-HCl, 10% SDS, 10% glycerol, 10% 2-mercaptoethanol and 2 mg/mL bromophenol blue) in a ratio of 1:1 and heated at 95 °C for 5 min. Pre-stained protein marker and 10 μL of each sample were loaded in each well on a Criterion^TM^ XT 4–12% gradient SDS-PAGE gel (Bio-Rad; 3450124) and electroblotted onto a nitrocellulose membrane (Bio-Rad) as detailed in our previous publications [33,38,47]. The membranes were blocked with 5% bovine serum albumin in Tris-buffered saline (pH 7.4) containing 1% Tween 20 (TBST) and then probed with primary antibodies against phosphorylated tau at Ser202/Thr205 (AT8; 1:1000 dilution), Ser396 (PHF13; 1:1000 dilution), Thr212/Ser214 (AT100; 1:1000 dilution) and housekeeping protein GAPDH (as a loading control; 1:100,000 dilution) overnight at 4 °C. Fluorescently labelled secondary antibodies were employed to generate immunoreactive signals, which were then detected on an Odyssey Infrared System (LI-COR Biosciences) at 700 and 800 nm. The immunoreactive signals of PHF13 and AT8 were analysed using ImageStudioLite (LI-COR Biosciences) and normalised by the corresponding GAPDH loading controls.

### 4.7. Immunohistochemistry

Immunohistochemistry was performed to assess the distribution of phosphorylated tau at Ser202/Thr305 in the brain of PS19 mice using the AT8 antibody. Serial coronal sections (30 µm) were cut from the brains of PS19 and WT mice at 2, 4, 6, 8 and 12 months of age on a cryostat (Leica CM 1950) at −20 °C. Sections were stored at −20 °C in cryoprotectant (30% *v*/*v* glycerol, 30% *v*/*v* ethyl glycol, 20% *v*/*v* 0.1 M phosphate buffer and 20% *v*/*v* H_2_O) until staining.

Unless stated, all immunohistochemical steps were performed at room temperature. Between each step, triplicate washes in phosphate buffered saline (PBS) were performed on an orbital shaker. The sections containing the frontal cortex, dorsal striatum, dorsal hippocampus and entorhinal cortex were selected from all animals and processed at the same time. To remove any aldehyde radicals from fixation and endogenous peroxidase activity, the sections were respectively incubated in 0.1 M glycine in PBS for 10 min and then 1% *v*/*v* hydrogen peroxidase (H_2_O_2_) and 40% *v*/*v* methanol in PBS for 30 min. The sections were then blocked with 10% *v*/*v* normal goat serum in blocking buffer (1% wt/vol bovine serum albumin and 0.3% *v*/*v* triton in PBS) for 1 h. Overnight, all sections were incubated with mouse AT8 primary antibody (1:1000 in blocking buffer) at 4 °C. All sections were then incubated in a biotinylated link solution containing biotin-labelled affinity isolated goat anti-mouse antibody for 90 min, followed by a 60 min incubation with a streptavidin/HRP complex solution. Phosphorylated tau Ser202/Thr205 (AT8) immunoreactivity was visualised as a brown colour due to the reaction with 3,3’-diaminobenzidine and urea/H_2_O_2_ tablets that were dissolved in 5 mL PBS. Sections were mounted on gelatine slides. The dried slides were put into a methanol series ending with two xylene incubations for dehydration and cover slipped with DPX mounting media. To confirm the specificity of the AT8 antibody, the primary antibody step was omitted from one section as a negative control. All images were captured using a Nikon microscope (Ti2E) mounted on a digital camera and NIS-Elements digital microscopy software.

### 4.8. Statistical Analyses

All data were analysed using two-way analysis of variance (ANOVA) followed by post hoc tests (uncorrected Fisher’s LSD) to determine the effects of genotype, age and their interaction. Statistical analyses were performed using GraphPad Prism software, and all data are presented as mean ± SEM. The level of significance was set at *p* < 0.05 for all comparisons.

## Data Availability

The data presented in this study are available in the article.
